# Bladder outlet obstruction disrupts circadian bladder function in mice

**DOI:** 10.1038/s41598-020-68499-w

**Published:** 2020-07-14

**Authors:** Takeya Kitta, Hiroki Chiba, Yukiko Kanno, Tsuyoshi Hattori, Madoka Higuchi, Mifuka Ouchi, Mio Togo, Yui Takahashi, Mai Michishita, Tatsuya Kitano, Nobuo Shinohara

**Affiliations:** 1 Department of Renal and Genitourinary Surgery, Graduate School of Medicine, Hokkaido University, Kita 15 Nishi 7, Kita-ku, Sapporo, Hokkaido 060-8638 Japan; 2 Department of Medical Affairs, Asahi Kasei Pharma Corporation, Tokyo, Japan; 3 Laboratory for Pharmacology, Pharmaceuticals Research Center, Asahi Kasei Pharma Corporation, Tokyo, Japan

**Keywords:** Neurophysiology, Urology

## Abstract

The circadian clock programs daily rhythms and coordinates multiple behavioural processes, including micturition. Partial bladder outlet obstruction (pBOO) in mice produces hyperactive voiding. However, long-term effects of pBOO on bladder function have not been clarified. In this study, we investigated micturition under conditions of impaired circadian bladder function by inducing long-term pBOO by tying the proximal urethra. Micturition behavior was evaluated at 1, 3, 6 and 12 months after surgery. We used automated voided stain on paper method for a precise micturition recording for mice. And quantitative assessment of gene expression was performed at 24 months after pBOO surgery using qRT-PCR procedure. The micturition frequencies in the pBOO group were significantly decreased at 3, 6, and 12 months compared to those at 1 month after operation in the same group (p < 0.05). Body weight of pBOO mice was significantly increased compared to sham operated mice at 12 months. The expression level of mRNA was exhibited a 3.4-fold nominal increased for a 5-HT2B receptor in the pBOO group compared to the sham group. The current study found that long-term pBOO led to disruption of the circadian bladder function (the day/night cycle) in mice, similar to those observed in human as nocturia. This disruption is possible involvement of the gain of body weight and/or serotonergic alteration after pBOO.

## Introduction

In men as a result of benign prostatic enlargement bladder outlet obstruction (BOO) is one of the most common causes of lower urinary tract symptoms (LUTS). Partial BOO (pBOO) significantly alters bladder morphology and function. Clinically, benign prostatic hyperplasia, bladder neck sclerosis, urethral valves or urethral strictures can cause mechanical BOO^1^. Although a correlation between LUTS and BOO or metabolic syndrome are known, to our knowledge, the detailed aetiology of LUTS remains unknown. Some symptoms, e.g. nocturia, remain to treat completely due to unclear mechanisms of LUTS. Several studies have shown that nocturia is a common complaint and (one of) the most frequent events in male LUTS. Nocturnal voiding disrupts sleep, and therefore repeated nocturnal voiding deteriorates the health-related quality of life^2^. There is very little information about the micturition behaviour as circadian bladder function in animal model.

Previous studies have shown that pBOO induces non-voiding contraction and shortening of inter-contraction interval in urinary bladder in animals^3,4^. Furthermore, pBOO leads to several morphological and functional changes in afferent pathways as well as in the bladder. To empty partial obstructed-bladder, pBOO produces compensate hypertrophy of detrusor, which causes gain of the thickness and weight of the bladder wall^5,6^. Although several subtypes of serotonergic and adrenergic receptors modulate the contractile response of the detrusor muscle^7–9^, to our knowledge no investigation has examined the role of these receptors in long-term BOO.

In vitro studies, BOO changes detrusor contractility using detrusor muscle isolated from experimental animal. In mice, pBOO produces bladder hypertrophy and hyperactive voiding in cystometrogram. The cystometry is one of the most helpful procedures on analysing functions of a urinary bladder but it cannot replicate physiological condition completely even if it performed under conscious condition.

Almost every life on earth is shaped by the 24-h rotation of our planet around its axes. The endogenous circadian clocks adapt daily rhythms and coordinate multiple behavioural and physiological processes, including micturition. At the molecular level, circadian clocks comprised a set of clock genes organized in a system of interlocked transcriptional-translational feedback loops. Recently, localization of mice bladder clocks controlling urination behaviour has been reported^10^.

Male LUTS may be provoked by metabolic abnormalities^11^. The chronic immune responses induced by acute and temporal physiological stresses generate injury, including ischemia^12^. To prevent organ injury, the body then attempts to provide an anti-inflammatory response, which can lead in turn to body weight gain. These processes can occur over the course of long time periods. However, long-term effects of pBOO have not sufficiently been clarified. In this study, we investigated the micturition behaviour as circadian bladder function of long-term pBOO in mice. And we focused on serotonergic and adrenergic receptors, if the behavioral changes occur, a post hoc analysis of the expression of the mRNAs encoding serotonergic and noradrenergic receptors will be performed.

## Results

During the current study, three mice died because of anaesthesia in the experimental course.

### Metabolic cage study

**Figure 1:**
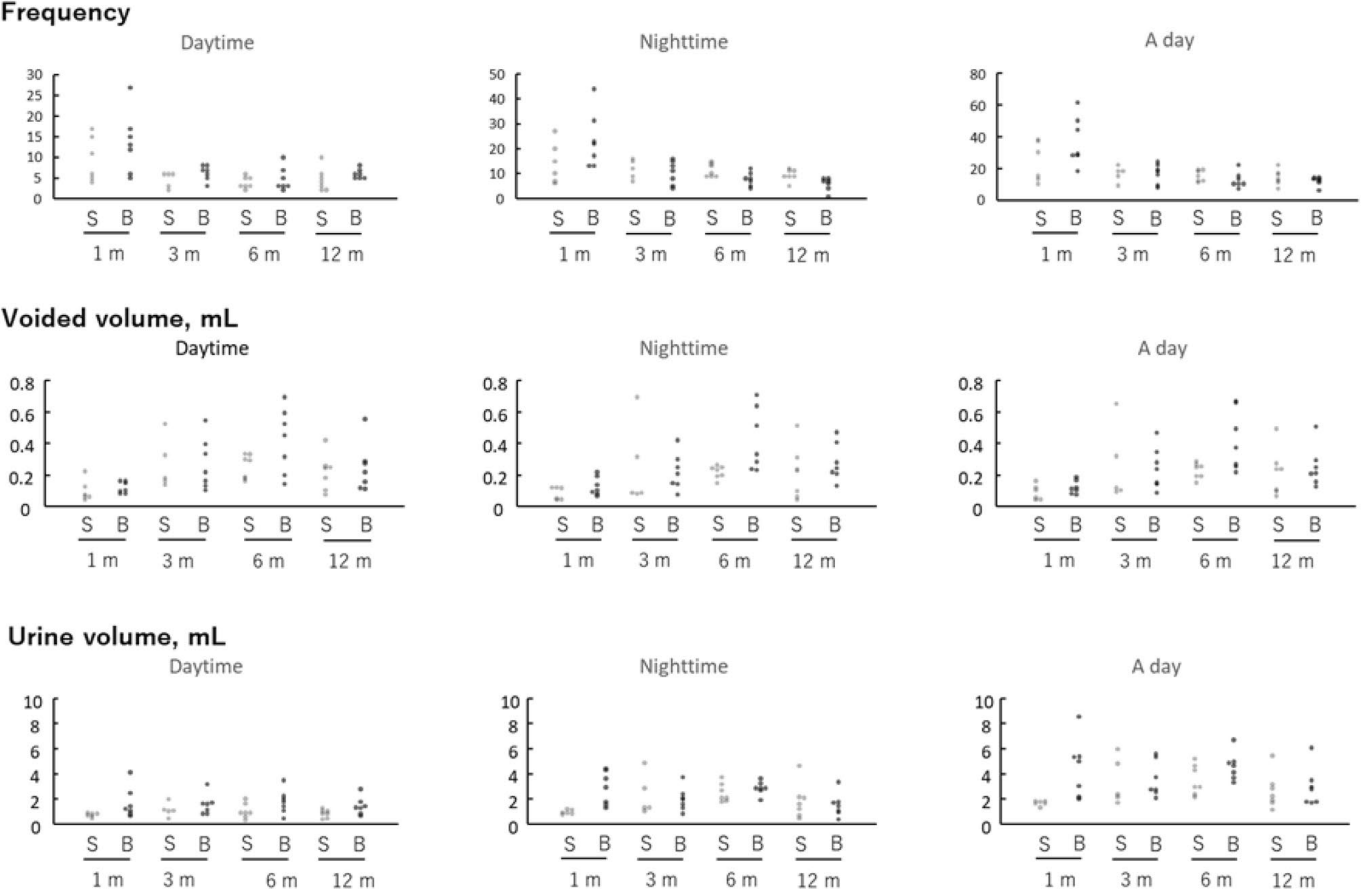
Scatter plots of the individual data obtained from the metabolic cage experiment. Frequency, voided volume, and urine volume are plotted separately. Light and dark gray circles indicate the sham group (S) and the pBOO group (B), respectively.

Three, 6 and 12 months after surgery, the total number of micturition, day time, and night time in the pBOO group was significantly decreased compared to pBOO mice 1 month after operation (^†^p < 0.05). In sham mice, the frequencies of nighttime micturition events were higher than in the daytime at 6 and 12 months. However, in BOO mice, those differences were no longer seen (circadian bladder function disturbance) (Table 1 and Fig. 1). There was a tendency for the increase in the body weight of pBOO mice compared to sham operated mice (27.4 g vs. 24.6 g in median) at 6 months after pBOO procedure. Twelve months after pBOO procedure, body weight of pBOO mice were significantly increased compared to sham operated mice (34.5 g vs. 25.3 g in median) (*p < 0.05) (Table 1). And, in pBOO mice, surrounding tissue of bladder adjacent was increased and tissue was adipose.

**Table 1:** Summary of results in the aVSOP (automated voided stain on paper) study.

Months	Sham				pBOO			
	n	Day	Night	Total	n	Day	Night	Total
**Frequency**								
1	6	9.7 ± 5.5 [3.9, 15.4]	14.2 ± 8.2 [5.6, 22.8]	23.8 ± 12.6 [10.6, 37.1]	7	13.6 ± 7.4 [6.7, 20.4]	23.3 ± 11.1 [13.0, 33.6]	36.9 ± 15.2 [22.8, 50.9]
3	5	4.6 ± 1.9 [2.2, 7.0]	11.8 ± 3.8 [7.0, 16.6]	16.4 ± 4.8 [10.4, 22.4]	7	6.3 ± 1.8^†^ [4.6, 7.9]	10.3 ± 4.8^†^ [5.9, 14.7]	16.6 ± 6.1^†^ [10.9, 22.2]
6	7	3.9 ± 1.5 [2.5, 5.2]	11.3 ± 2.6 [8.9, 13.7]	15.1 ± 3.5 [11.9, 18.4]	7	4.7 ± 2.9^†^ [2.1, 7.4]	7.7 ± 2.8^†^ [5.2, 10.3]	12.4 ± 4.9^†^ [7.9, 17.0]
12	7	4.6 ± 2.8 [2.0, 7.2]	9.4 ± 2.3 [7.3, 11.6]	14.0 ± 4.8 [9.5, 18.5]	7	6.0 ± 1.2^†^ [4.9, 7.1]	5.9 ± 2.5^†^ [3.5, 8.2]	11.9 ± 2.8^†^ [9.3, 14.4]
**Total micturition volume (μL)**								
1	6	727.9 ± 161.8 [558.1, 897.6]	950.3 ± 186.5 [754.6, 1,146.1]	1,678.2 ± 200.1 [1,468.2, 1,888.2]	7	1,667.9 ± 1,220.1 [539.5, 2,796.3]	2,823.8 ± 1,326.3 [1,597.2, 4,050.4]	4,491.7 ± 2,300.4 [2,364.2, 6,619.2]
3	5	1,103.1 ± 550.3 [419.8, 1,786.5]	2,277.0 ± 1,616.7 [269.6, 4,284.4]	3,380.1 ± 1,864.2 [1,065.5, 5,694.8]	7	1,544.2 ± 806.2 [798.6, 2,289.9]	1,976.5 ± 932.4 [1,114.2, 2,838.8]	3,520.7 ± 1,394.4 [2,231.2, 4,810.3]
6	7	1,032.9 ± 596.4 [481.3, 1,584.4]	2,473.9 ± 747.1 [1,782.9, 3,164.8]	3,506.7 ± 1,193.8 [2,402.7, 4,610.8]	7	1,754.9 ± 968.5 [859.2, 2,650.6]	2,840.4 ± 523.8 [2,356.0, 3,324.9]	4,595.3 ± 1,102.8 [3,575.4, 5,615.2]
12	7	813.8 ± 307.9 [529.1, 1,098.6]	1,810.5 ± 1,385.4 [529.2, 3,091.8]	2,624.3 ± 1,410.9 [1,319.5, 3,929.2]	7	1,409.0 ± 707.8 [754.4, 2,063.6]	1,526.5 ± 917.6 [677.8, 2,375.1]	2,935.4 ± 1,556.5 [1,495.9, 4,375.0]
**Average voided volume (μL)**								
1	6	99.9 ± 68.5 [27.9, 171.8]	85.6 ± 40.6 [42.9, 128.2]	90.2 ± 48.2 [39.6, 140.9]	7	120.8 ± 36.5 [87.0, 154.6]	129.3 ± 57.9 [75.7, 182.8]	122.8 ± 43.0 [83.0, 162.6]
3	5	267.3 ± 159.5 [69.3, 465.3]	253.8 ± 266.6 [− 77.2, 584.7]	259.5 ± 240.8 [− 39.5, 558.5]	7	271.6 ± 162.1 [121.6, 421.6]	221.5 ± 114.1 [116.0, 327.1]	245.5 ± 132.7 [122.7, 368.2]
6	7	255.6 ± 75.9 [185.5, 325.8]	219.7 ± 40.4 [182.3, 257.0]	230.1 ± 48.8 [185.0, 275.3]	7	417.7 ± 206.1 [227.1, 608.2]	421.9 ± 197.3 [239.4, 604.4]	419.8 ± 190.2 [243.9, 595.7]
12	7	219.8 ± 114.5 [113.9, 325.8]	212.9 ± 165.5 [59.8, 365.9]	217.0 ± 146.8 [81.2, 352.8]	7	247.1 ± 152.0 [106.5, 387.7]	281.3 ± 118.9 [171.3, 391.3]	252.6 ± 124.8 [137.2, 368.0]
**Body weight (g)**								
1	6			19.5 ± 1.5 [17.9, 21.1]	7			20.4 ± 1.7 [18.8, 22.0]
3	5			22.4 ± 2.8 [18.0, 26.8]	7			25.2 ± 2.8 [22.7, 27.8]
6	7			24.6 ± 1.2 [23.5, 25.7]	7			27.4 ± 4.6 [23.1, 31.7]
12	7			25.3 ± 2.1 [23.4, 27.2]	7			34.5 ± 6.2* [28.8, 40.3]

Data are presented as mean ± standard deviation; the 95% confidence intervals are shown in brackets.

*pBOO* partial bladder outlet obstruction.

*p < 0.05 vs. sham group at the respective month by two-tailed non-paired Student’s *t* test.

^†^p < 0.05 vs. 1-month group by two-tailed paired Student’s *t* test.

### RT-PCR and bladder weight at 2 years after the BOO creation

At 2 years after the BOO creation, mean weight of urinary bladders were 34.5 ± 3.0 mg (n = 4) and 38.3 ± 14.3 mg (n = 7) in the sham and BOO groups, respectively. The result of expression levels of mRNA for several receptors was shown in Table 2. Any mRNA tended to express higher in BOO rats than in sham rats. The expression levels of them were 5-HT2B receptor > 5-HT2C receptor > 5-HT2A receptor > α1A adrenoceptor > α1D adrenoceptor > 5-HT4 receptor = 5-HT7 receptor, β1 adrenoceptor, β3 adrenoceptor in a descending order. The expression level of β2 adrenoceptor was no changed.

**Table 2:** The relative expression levels of RT-PCR for urinary bladder in BOO mice.

Receptor	Sham, n = 4			pBOO, n = 7		
	Mean	Standard deviation		Mean	Standard deviation	
		Positive	Negative		Positive	Negative
5-HT2A	1.000	0.177	0.150	1.462	0.287	0.240
5-HT2B	1.000	0.820	0.450	3.407	2.988	1.592
5-HT2C	1.000	1.085	0.520	1.699	1.340	0.749
5-HT4	1.000	0.336	0.251	1.342	0.295	0.242
5-HT7	1.000	0.516	0.341	1.442	0.692	0.467
α1A	1.000	0.245	0.197	1.666	1.039	0.640
α1D	1.000	0.561	0.359	1.561	0.889	0.567
β1	1.000	0.242	0.195	1.350	0.221	0.190
β2	1.000	0.465	0.317	0.947	0.164	0.140
β3	1.000	0.279	0.218	1.402	0.272	0.227

The expression levels in the pBOO group were shown as ratio with reference to the sham group.

*pBOO* bladder outlet obstruction, *RT-PCR* real-time polymerase chain reaction.

## Discussion

Long-term pBOO impairs the circadian bladder function (the day/night cycle) in mice. In mammals, micturition frequency during the sleep period is lower than that during the awake period. Since murine animals are nocturnal, mice have more frequent rhythm for micturition during the sleep phase than during the day. Previous studies have reported that animals with pBOO showed various changes in the lower urinary tract functions. To the best of our knowledge, the study of Murakami et al.^13^ is the longest study of pBOO using rat model as 6 months. Our group reported that pBOO leads to several morphological and functional changes, which need certain period^6^. However, it currently remains unclear whether how long needed remodelling and change the function of lower urinary tract. Therefore, the aim of this study was to determine the effects of 12 months effect of pBOO using mice.

Assessing pattern of micturition in mice is challenging^14^. Because even in adult mice the urine volume voided per micturition is too small, it was not until development of the automated voided stain on paper method that the mice micturition had been assessed continuously and accurately. The fine procedure was devised to record the micturition of a mouse with free moving including an access to food and water for experiment days^15^. By this procedure, physiological voiding behaviour, e.g., urine volume and frequency, came to be able to measure finely and easy. Negoro et al. also reported that the establishment of a day-night rhythm of adult mice from neonate. This is a key axis for analysing micturition behaviour since the investigation of such rhythms may be helpful to translate into improvement of paediatric nocturnal enuresis or adult nocturia. A murine cystometrogram indicated that pBOO produces bladder hypertrophy and hyperactive voiding^6^. The cystometrogram is one of the most helpful procedures to measure functions of a urinary bladder, but it cannot replicate physiological condition anaesthesia may affect bladder capacity and diurnal behaviour^16^. In humans and rodents, functional bladder capacity and rate of urine production during the sleep period are increased and decreased, respectively. By which, they are split off from waking and their sleeping is protected^17^. The aVSOP system, which has been developed to mimic a frequency-volume chart at a human clinic, is an excellent translational procedure.

**Figure 2:**
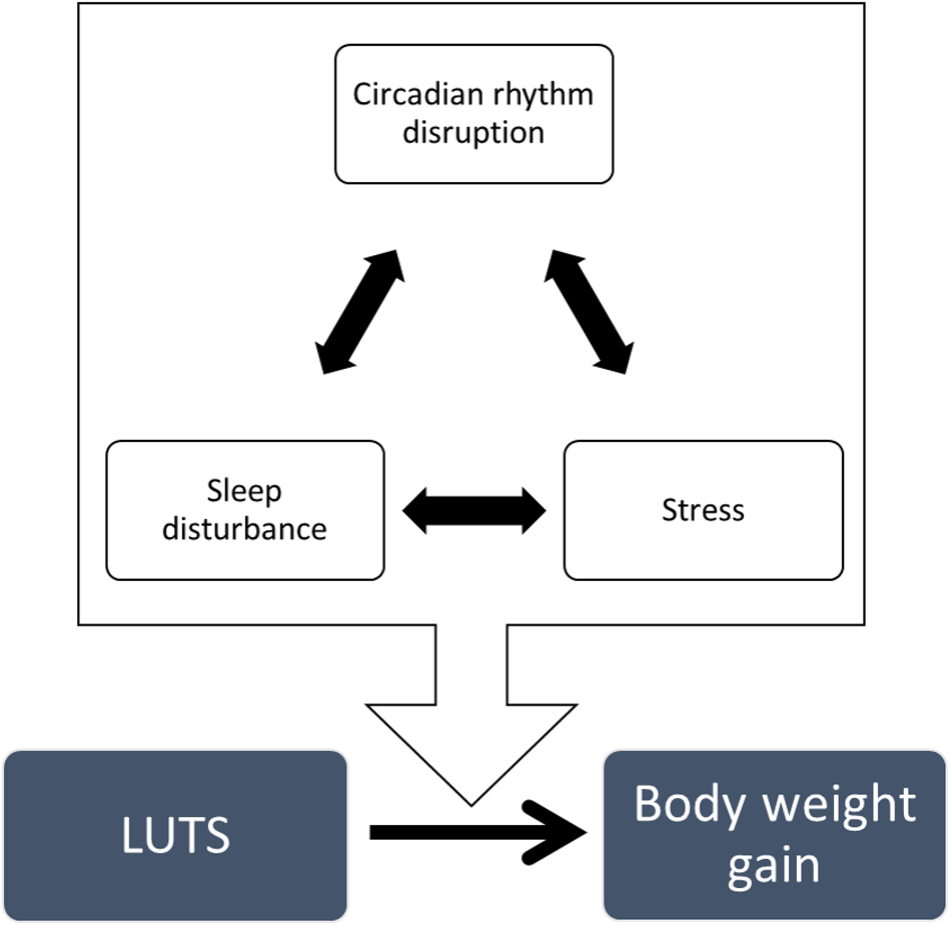
A scheme of the interaction of stress, sleep disturbance, and circadian rhythm disruption promotes body weight gain. Sleep and circadian rhythm reciprocally modulate the hypothalamus–pituitary–adrenal (HPA) axis in normal conditions; however, once chronic stress provokes, followed by the HPA axis constantly activating. Finally, the control of circadian rhythm goes wrong and the rhythm is disrupted. When these interconnected relationships are dysfunctional, the relations will be led to a perpetual negative spiral that synergistically exacerbates pathophysiologic changes in each other. *LUTS* lower urinary tract symptoms.

The increase in day time micturition frequency and the decrease in average voided volume in day time were observed from 6 months after surgery. And 12 months after pBOO procedure, body weight was significantly increased compared to sham operated mice. In the current study, 6 months or later after pBOO surgery, during harvest bladder, adjacent soft tissue was increased and tissue was adipose. Asplund reported that the increase body weight induced the increasing number of nocturnal voiding. Although the details of mechanisms are remained to be cleared, frequent nocturnal micturition increases the risk of obesity, as could be related the negative impact on sleep^18^. Recently, both of male and female suffered from LUTS will be overweight and will have features of the metabolic syndrome (Mets). High-Fat diet attenuates amplitude of clock gene expression^19^. And, increasing body weight, Leptin, which is produced by adipose tissue, controls the major CNS systems that modulate food intake and energy expenditure^20^. From these perspectives, increase in the body weight of pBOO mice could disrupts circadian bladder function. However, there is no report about the concept that LUTS induce Mets. In the present study, long-term bothersome of LUTS may raise undesirable psychiatric responses, e.g. anxiety, depressive disorders, and hyperorexia. Corticotropin-releasing factor (CRF), which has been identified as a neuropeptide responsible for initiating many of the endocrine, autonomic and behavioural responses to stress, possibly plays a key role. Interestingly, stress, sleep disturbance, and circadian rhythm disruption reciprocally connect in molecular signaling and anatomic pathways. The hypothalamus–pituitary–adrenal (HPA) axis, which is the main pathway of stress response, is regulated by the circadian rhythm^21^. Psychological stressors also modulate cortisol levels, which is elevated together with the increment of the stress response by circadian misalignment and sleep restriction. Circadian misalignment induces sleep disruption and alters cortisol production^22^. Stress, circadian rhythms, and sleep all work each other (Fig. 2).

Stress, sleep disturbance, and circadian disruption interact at many levels to affect metabolic disruption. Although the body weight markedly gained in the pBOO group as compared to the sham group (Table 1), to clarify, this mechanism we have to confirm the amount of adipose tissue and the plasma leptin concentrations. However, it is difficult to quantify the amount of adipose tissue especially mice model. This quantification of Mets in small animals to define should be a future issue.

**Figure 3:**
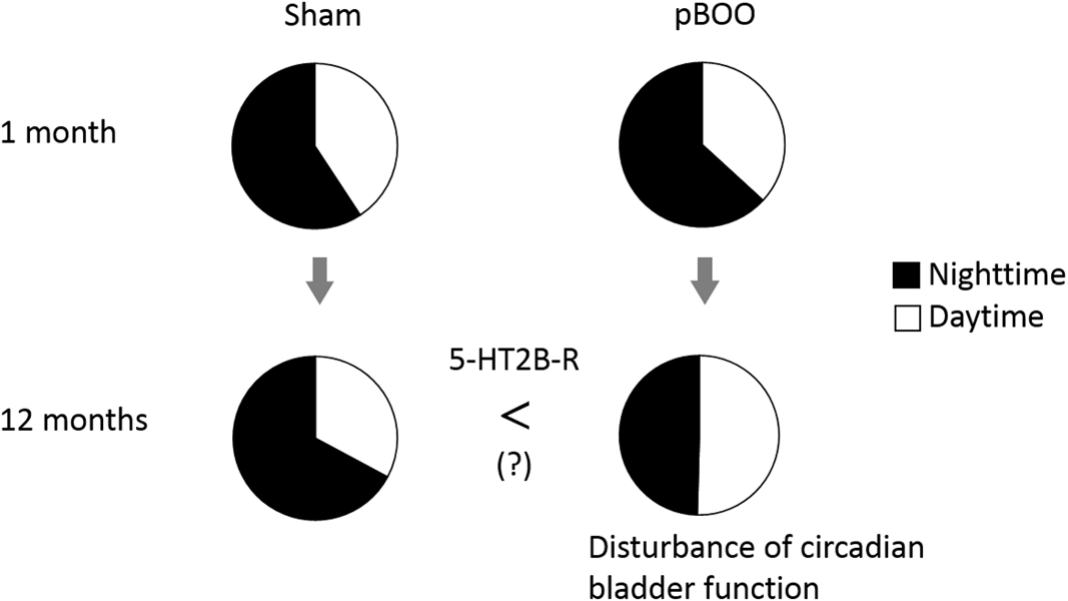
Circadian bladder function in point of the micturition rate for 24 h. Circles show the mean total-micturition for 24 h. Areas of black and white reveal percentages of micturition in night and day time, respectively. *5-HT2B-R* 5-Hydroxytryptamine 2B receptor

In the present study, the result of RT-PCR was distinguishingly tended to be high for all receptors, particularly a 5-HT2B receptor, except for β2-adrenoceptor in the pBOO group. In the previous study, ketanserin, 5-HT2A/2C receptor antagonist, attenuates detrusor contraction in vitro^7^. Recently, it was reported that the 5-HT2B receptor is associated with the serotonin-evoked contraction of detrusor strips which is isolated from pBOO rats after 1-week operation, and the detrusor intensify a 5-HT evoked contraction in pBOO rats as compared to sham rats^23^. In addition, 5-HT2A and 5-HT2B receptor subtypes contribute the contraction at high and low concentration of serotonin, respectively^23^. Therefore, the present result of overexpression of 5-HT2B receptor suggests that the detrusor easily contracts at a low concentration of serotonin in the pBOO group as compared to the sham group at 2-year after operation. This mention may indicate that a high contractility needs in an obstructed urinary bladder at the old age (Fig. 3). Although further experiments are essential, the long-term obstruction may transform the bladder contractility from overactive to underactive, particularly after 12 months and more from urinary obstruction.

In the last decade much attention has focused on the role of the sympathetic nervous system and α1-adrenergic receptors (α1-ARs) in the dynamic (smooth muscle contraction) component of pBOO. In regard to changes in α1-AR subtype expression following pBOO, Hampel et al. observed in a rat model of pBOO that expression level of the α1d-AR mRNA was three times greater than the sham group after 6 weeks of obstruction^24^. The present results indicated the same trend but were mild increase. The difference between the previous investigation and the present result possibly shows transforming from high frequency at 6 weeks to eliminated frequency at 12 months.

This study has some limitations. The quantification of Mets in mice is a subject for future analysis. And, the main focus of the present study is the experiment of metabolic cage, and RT-PCR is secondary carried out. Although a mismatch of timing of measurement for levels of mRNA is evident limitation, an interpretation can be understood as the long-term results over 12 months.

This is the first study to demonstrate the chronological change of circadian bladder function and body weight change in long-term (12 months) pBOO mice. The current study has found that pBOO lead to disruption of one of the circadian bladder functions (the day/night cycle) in mice, similar to those observed in human as nocturia.

## Methods

### Animals and study design

Eight-week-old female C57/BL6 mice were housed in a room maintained at 20–26 °C and 35–75% relative humidity, which in the facility is generally stable at approximately 60%, with an alternating 12 h light (from 7 a.m.)/dark cycle (from 7 p.m.). All mice were allowed free access to food pellets and tap water. The study was conducted in compliance with the Internal Regulations on Animal Experiments at the Institutional Committee. The proximal urethra was tied as in the procedure for pBOO as previously described^6^. In brief, “The bladder and proximal urethra were exposed through a lower abdominal incision. The proximal urethra was freed from the vaginal wall by careful dissection to avoid injury to the periurethral blood vessels. To create an infravesical obstruction, a metal rod with a diameter of 0.55 mm was placed beside the proximal urethra and a 4-0 silk ligature was tied around the urethra and metal rod. The rod was subsequently removed and the abdominal incision was closed.” Sham surgery was performed. Micturition behaviour was evaluated at 1 month, 3 months, 6 and 12 months after pBOO surgery. Additionally, to explore the mechanism on the changes of micturition, quantitative real-time polymerase chain reaction (qRT-PCR) was conducted at 24 months. Animals were treated in accordance with the National Institutes of Health animal care guidelines. All experiments, including protocols, were approved by Institutional Animal Care and Use Committee of National University Corporation Hokkaido University (No. 16-0103).

### Metabolic cage study

Each mouse was placed in an individual metabolic cage at 1, 3, 6 and 12 months after pBOO and sham surgeries. We used aVSOP (automated voided stain on paper) method^13^, which is a precise micturition recording system for mice. Urine stains were counted and traced, ranging from 10 to 800 μl^25^. Each void was captured by placing, at the base of the cage, a sheet of paper that was rolled up with a laminated filter and pre-treated to turn the edge of urine stains deep purple. The paper was wound up at a speed of 10 cm/h under a water-repellent wire lattice. Urine stains were counted and traced, and stain sizes were converted to micturition volume by using the formula of a standard curve. The standard curve was generated by dropping normal saline (in volumes of 10–800 μL) onto similarly treated paper and tracing the resulting stained areas. To relieve stress, the mice were provided with free access to water and food while housed in the individual metabolic cages. To reduce voiding activity that might result from any stress, the animals were habituated to the metabolic cages for 24 h before initiating measurement of the voiding events. Mice were placed into individual metabolic cages for 24 h and voids were captured with paper at the base of the cage. The parameters evaluated were voided volume and time per void, total urination frequency (daytime and night time) and total urine volume.

### Tissue homogenization

A bladder tissue was cut into several specimens of 20 mg or less. Individually, the specimen was each transferred into a 2 mL-tube containing 4.8 mm ϕ of a bead made from stainless steel and a lysis buffer. Homogenization was performed in the tube for ten runs of 30 s each at 3,500 rpm using Micro smash™ (MS-100R; TOMY SEIKO, Tokyo, Japan). The tube was cooled at 4 °C for 30 s between the runs.

### Quantitative RT-PCR

Quantitative assessment of gene expression was performed using qRT-PCR according to the manufacturer’s manual. RNA was extracted from the urinary bladder using a RNeasy Fibrous Tissue Mini Kit (#74704; QIAGEN, Venlo, Netherlands). Purified RNA was reversely transcribed cDNA using a SuperScript VILO cDNA Sythesis Kit (#11754-050; Invitrogen, Massachusetts, USA). The experimental procedure of each kit was carried out according to the manufacturer’s instructions. Quantitative real-time polymerase chain reaction was performed using Quant Studio 7 Flex (#4484643; Applied Biosystems, Massachusetts, USA) with Taqman (Applied Biosystems, Massachusetts, USA) and THUNDERBIRD (#QPS-101; TOYOBO, Osaka, Japan). RPL 19 was applied as housekeeping gene. Relative expression level of each mRNA was then normalized to the expression level of RPL 19, using the 2^−ΔΔCT^ method.

### Statistical analysis

Statistical analyses were conducted with the GraphPad Prism for Windows (GraphPad Software, San Diego, CA, USA). All data are expressed as mean ± standard deviation. Student’s t test was used to compare the metabolic cage parameters pBOO mice to sham operated mice. Dunnett’s test was performed to compare the values at each month after pBOO surgery with referring to the value at 1 month. P values < 0.05 were statistically significant. Regarding qRT-PCR, the mean of the pBOO group is shown as ratio referred to the sham group.

## Abbreviations

BOO: Bladder outlet obstruction
pBOO: Partial bladder outlet obstruction
LUTS: Lower urinary tract symptoms
aVSOP: Automated voided stain on paper
Mets: Metabolic syndrome
CRF: Corticotropin-releasing factor
HPA: Hypothalamus–pituitary–adrenal
α1ARs: α1-Adrenergic receptors
